# Zinc toxicity response in *Ceratoides arborescens* and identification of *CaMTP*, a novel zinc transporter

**DOI:** 10.3389/fpls.2022.976311

**Published:** 2022-09-06

**Authors:** Xingyue Li, Lin Zhang, Haiyan Ren, Xiaoyu Wang, Fugui Mi

**Affiliations:** ^1^College of Grassland, Resources and Environment, Inner Mongolia Agricultural University, Hohhot, China; ^2^M-Grass Ecology and Environment (Group) Co., Ltd., Hohhot, China

**Keywords:** zinc deficiency, zinc excessive, photosynthesis, peroxisome, transport, endoplasmic reticulum

## Abstract

Zinc (Zn) is an essential micronutrient for several physiological and biochemical processes. Changes in soil Zn levels can negatively affect plant physiology. Although the mechanism of Zn nutrition has been studied extensively in crops and model plants, there has been little research on steppe plants, particularly live in alkaline soils of arid and semiarid regions. *Ceratoides arborescens* is used in arid and semiarid regions as forage and ecological restoration germplasm, which is studied can enrich the mechanism of Zn nutrition. The plants were exposed to three different Zn treatments, Zn-deficient (-Zn 0 mM L^−1^), Zn-normal (Control, 0.015 mM L^−1^), and Zn-excess (+Zn, 0.15 mM L^−1^), for 3 weeks. Individual biomass, ion concentrations, photosynthetic system, and antioxidant characteristics were measured. High Zn supply significantly decreased plant biomass and induced chlorosis and growth defects and increased Zn concentration but decreased Fe and Ca concentrations, unlike in controls (*p* < 0.05). High Zn supply also reduced plant chlorophyll content, which consequently decreased the photosynthesis rate. Increased concentrations of malondialdehyde and soluble sugar and activities of peroxidase and superoxide dismutase could resist the high-level Zn stress. In contrast, low Zn supply did not affect plant growth performance. We also identified a novel protein through RNA transcriptome analysis, named *CaMTP*, that complemented the sensitivity of a yeast mutant to excessive Zn, which was found to be localized to the endoplasmic reticulum through transient gene expression in Nicotiana benthamiana. The gene *CaMTP* identified to be highly sensitive to Zn stress is a potential candidate for overcoming mineral stress in dicot crop plants.

## Introduction

Zn, the second most abundant transition metal in living organisms, is vital to crop nutrition as it is required in various enzymatic reactions, metabolic processes, and oxidation reduction reactions (Broadley et al., 2007; Al Jabri et al., 2022). Zn is one of the most limited micronutrients in soils and plants (Amini et al., 2022). With high pH or alkaline stress, many arid and semiarid lands are Zn deficient (McFarland et al., 1990; Scharwies and Dinneny, 2019). Zn deficiency could lead to a lack of cellular integrity, dwarf stems, light chlorosis, and decreases in chlorophyll synthesis (Khatun et al., 2018; Kabir et al., 2021). The deficiency of Zn also causes the accumulation of excessive reactive oxygen species (ROS) that may be due to the lower concentration of the Cu-Zn-SOD enzyme (Marreiro et al., 2017). Another effect of Zn deficiency is reduced plant pigments and interference with the process of photosynthesis, thereby inhibiting plant growth (Sidhu et al., 2020).

Anthropogenic activities, including mining, smelting, and fertilizing with sewage sludge, are associated with Zn buildup in soils and water, resulting in Zn pollution (Broadley et al., 2007; Sinclair and Krämer, 2012). High Zn levels may also induce toxicity affecting most vital processes in plants (Marichali et al., 2016). Common symptoms of Zn toxicity include growth inhibition, repression of root elongation owing to the inhibition of cell proliferation, alteration in water and nutrient uptake, loss of membrane integrity, disruption of redox homeostasis, reduction of chlorophyll content and subsequent photosynthesis, generation of ROS, and the manifestation of oxidative stress (Balafrej et al., 2020). The induction of the antioxidant system has been described as a putative strategy against trace metal toxicity; accumulating evidence indicates that the activities of catalase (CAT), superoxide dismutase (SOD), guaiacol peroxidase (GPX), and ascorbate peroxidase (APX) are increased in Zn-exposed plants (Rout and Das, 2003; Ahmad et al., 2022).

Plants have established a tightly controlled system to balance the uptake, utilization, and storage of Zn ions (Yin et al., 2022). One mechanism involves the production of root exudates to tolerate deficiency or excess (Santi and Schmidt, 2009; Tsednee et al., 2014). Furthermore, enzymes with antioxidant properties play key roles in controlling radicals (superoxide radical, hydrogen radical, and hydroxyl radical) and peroxides at the cellular level and protecting plants from abiotic stresses (Rout and Das, 2003; Kumar Tewari et al., 2008; Kabir et al., 2021). At the molecular level, Zn homeostasis in plants is tightly regulated by Zn sensors and metal chelators involved in Zn acquisition and sequestration (Yin et al., 2022). Zn cannot diffuse across cell membranes; hence specific Zn transporters are required to transport Zn into the cytoplasm (Kaur and Garg, 2021). Some plant membrane transporter families, such as ZRT/IRT-like proteins (ZIP) family, P-type ATPase family, natural resistance-associated macrophage protein (NRAMP) family, and cation diffusion facilitator (CDF) family, have been shown to participate in the Zn^2+^ uptake, transport, and maintaining homeostasis (Krämer et al., 2007). CDF, also referred to as metal tolerance/transport protein (MTP) in plants, is involved in metal transport out of the cytoplasm and is induced by excess Zn (Ricachenevsky et al., 2013). MTP3 is located on the tonoplast (Zhao et al., 2018) and complements the yeast mutant zrc1cot1, which is sensitive to excess Zn levels (Lu et al., 2009), while MTP2 is located on the endoplasmic reticulum (ER) membrane (Sinclair et al., 2018), and MTP5 and MTP12 are located at Golgi in *Arabidopsis thaliana* and cucumber (Migocka et al., 2018).

The magnitude of Zn tolerance in plants depends on different plant species (Mateos-Naranjo et al., 2014), and the physiological range between deficiency and toxicity of Zn is narrow (Glińska et al., 2016). Many species grew in acidic soils since they are rich in metals (Meyer et al., 2016). A study with the establishment of salt-tolerant plants on soils contaminated with soluble salts by petroleum exploration activities in arid and semiarid areas showed that the survival rates of *Ceratoides lunata* and *Kochia prostrata* were 61 and 40%, respectively, but *Atripkx nummukzria* suffered 100% mortality (McFarland et al., 1990). This fact indicates that *Ceratoides* sp. can tolerate salt, but little is known regarding its response to Zn deficiency and toxicity. *Ceratoides arborescens*, a forage endemic to China, was selected in this study because it accumulates mineral elements in adequate capacities, produces copious amounts of high-quality biomass, and is widely used in arid and semiarid regions of China as an excellent forage and ecological restoration grass (Liu and Yi, 2004).

To date, data on the uptake or accumulation of nutrient elements and metabolic profiling of Zn stress in *C. arborescens* remain limited. Therefore, this study investigated the effects of Zn treatment on *C. arborescens* by analyzing plant growth, contents of ions and chlorophylls, antioxidant activities, and the photosynthetic system within a controlled hydroponic culture and identified the transcripts encoding proteins involved in Zn transport through yeast complementation. Our study is essential for bioaugmentation in arid and semiarid regions of dicot crop plants and elucidates the physiological and molecular mechanisms underlying the differential responses to various Zn supply levels in *C. arborescens*.

## Materials and methods

### Plant materials and cultivation technique

In October 2020, diaspores of *C. arborescens* were collected from the desert steppe of Siziwang Banner, Inner Mongolia, China (E 112.113942°, N 41.996905°). Dry seeds were stored at 4°C before being used in the germination experiment.

Seeds were sterilized with 75% ethyl alcohol for 3 min, followed by double Milli-Q water before being transferred to the germination tray in a growth chamber with a 12/12 h light/dark light period and 100–120 μmol m^−2^ S^−1^ light intensity. Sprouted three-day homogeneous plantlets were transferred to a hydroponic solution of pH 5.7 and cultured for 2 weeks.

The composition of the hydroponic solution (μM L^−1^) was as follows: KNO_3_ (5,000 μM L^−1^), CaCl_2_ (1,000 μM L^−1^), H_3_PO_4_ (1,000 μM L^−1^), MgSO_4_.7H_2_O (1,000 μM L^−1^), KI (2.5 μM L^−1^), H_3_BO_3_ (50 μM L^−1^), Fe-EDTA (50 μM L^−1^), MnSO_4_·H_2_O (50 μM L^−1^), ZnSO_4_·7H_2_O (15 μM L^−1^), Na_2_MoO_4_.2H_2_O (0.52 μM L^−1^), CoCl_2_·6H_2_O (0.053 μM L^−1^), and CuSO_4_·5H_2_O (0.05 μM L^−1^). Then, the seedlings were transferred to the following treatment solutions (pH 5.7) and cultured for 3 weeks: Zn- excess (+Zn), ZnSO_4_·7H_2_O (150 μM L^−1^); Zn-normal (control), ZnSO_4_·7H_2_O (15 μM L^−1^); and Zn-deficient (-Zn), ZnSO_4_·7H_2_O (0 μM L^−1^) (Wang et al., 2017).

### Metal contents analysis

Tissues from the root, stem, and leaf were harvested and dried at 105°C in an oven for 3 days, and then the Zn, Fe, and Ca levels were measured. The dried tissues were weighed and ground to a powder, which was later extracted with 5% HNO_3_ for 3 days. The extracts were centrifuged at 12,000 × *g* for 30 min, and the supernatants obtained were then filtered through a 0.45 μm membrane. The levels of Zn, Fe, and Ca were quantified using an Inductive Coupled Plasma Emission Spectrometer (ICP-OES_PQ9000; Analytikjena, German).

### Chlorophyll contents and photosynthesis activity parameters analysis

Plant samples, weighing 0.2 g, were grounded with liquid nitrogen and then extracted with 10 ml of 96% ethanol for 24 h in darkness; all these steps were carried out at 4°C. The extract was centrifuged at 10000 × *g* for 10 min, the absorbance of the supernatant was measured at 649 and 665 nm, and chlorophyll concentrations were calculated (van Dijk and Roelofs, 1988). The photosynthetic activity parameters, such as the quantum yield of photosystem I [Y(I)], the quantum yield of photosystem II [Y(II)], and the non-photochemical quenching (NPQ) levels were measured (photosynthesis measurement system GFS-3000; WALZ, German).

### Antioxidant enzyme activities and lipid peroxidation analysis

Approximately 0.5 g of fresh root tissues were grounded using liquid nitrogen, followed by homogenization in 1.5 ml of an extraction buffer consisting of 0.05 M pre-cooled phosphate buffer (pH 7.8). The extract was centrifuged at 10,000 × *g* for 20 min at 4°C, and the supernatant was collected and stored at −20°C. SOD activity was determined spectrophotometrically at a wavelength of 560 nm based on the inhibition of nitroblue tetrazolium (NBT) photochemical reduction (Giannopolitis and Ries, 1977). Peroxidase (POD) activity was assessed by determining guaiacol oxidation by H_2_O_2_ at a wavelength of 470 nm (Giannopolitis and Ries, 1977).

Approximately 0.5 g of fresh roots or shoots were homogenized with 5 ml of 5% (v/v) trichloroacetic acid (TCA) and then centrifuged at 10,000 × *g* for 10 min. MDA concentrations were determined by using the following formula (Hodges et al., 1999):


$$MDA\;\left(\mathrm{\mu mol}/g\;FW\right)=6.45\times \left(OD_{532}-OD_{600}\right)-0.56\times OD_{450}$$

Approximately 0.5 g of fresh root tissues were grounded in quartz sand and distilled water, followed by homogenization in 10 ml of distilled water and then heated at 80°C for 30 min. The homogenate was filtered, the filtrate volume was made up to 100 ml, and the soluble sugar (SS) levels were measured using resorcinol colorimetry (Williams and Martin, 1967).

### Gene expression and bioinformatics analysis

Roots of *C. arborescens* grown in Zn-excess conditions and control hydroponic solutions were collected and subjected to RNA extraction using the RNAiso for Polysaccharide-Rich Plant Tissue kit (Takara, China). Transcriptome analysis was conducted by Beijing Capital Bio Corporation according to the standard procedure of the Illumina HiSeq 2,500 sequencing platform. Transcripts encoding putative Zn^2+^ transporting proteins were identified by searching the transcript annotation tables for the keywords Zinc, Zn^2+^, Zinc transporting, Zn^2+^ transporting, ZIP, and CDF. *CaMTP* homolog was searched using its amino acid sequence at NCBI Protein Blast.^1^ The protein structure was predicated based on its amino acid sequence.^2^

### Subcellular localization of *CaMTP*

*CaMTP* was inserted into the vector pUC57 between the NotI restriction enzyme site on one side and the NotI and KpnI restriction enzyme sites on the other side of the vector. The full-length coding sequence of *CaMTP* without the stop codon was amplified from pUC57 using PrimeStar HS DNA Polymerase (Takara) and the primers “*CaMTP*-pENTR 3C-F” and “*CaMTP*-pENTR 3C-R” (Table 1) containing BamHI and NotI restriction enzyme sites, respectively. The PCR product was ligated into the pEASY-Blunt Simple Cloning Vector (TransGen, Beijing, China) and confirmed *via* sequencing. The *CaMTP* coding sequence was ligated between the BamHI and NotI sites of pENTR 3C (Thermo Fisher, United States). Subsequently, the *CaMTP* coding sequence without the stop codon was ligated downstream of the 35S promoter and upstream of the GFP coding sequence in plant binary expression vector pGWB605 (Nakagawa et al., 2007) using Gateway LR Clonase (Thermo Fisher). The pGWB605 vector harboring 35S::*CaMTP*-GFP fragment was introduced into Agrobacterium strain GV3101.

**Table 1:** Sequence of primers used to insert *CaMTP* into the pENTR 3C and pYUL2 vectors.

Primer name	Primer sequences (5′-3′)
*CaMTP*-pENTR 3C-F	CGCG GATC CATG TTT ACA GCC CGA CAT GAT CCC ACA C
*CaMTP*-pENTR 3C-R	TT GC GGCC GCGGAGC CCA ACG TCC AAC GAC AGA CAT
*CaMTP*-pYUL2-F	CGCG GATC CATG TTT ACA GCC CGA CAT GAT CCC ACA C
*CaMTP*-pYUL2-R	CCCA AGCT TTTA AGC CCA ACG TCC AAC GAC AGA CAT

Agrobacterium cells containing pGWB605-35S::*CaMTP*-GFP or individual subcellular marker vectors were co-infiltrated into the lower epidermis of tobacco (N benthamiana) leaves. Confocal microscopy (LSM 710; Zeiss, China) or super-resolution confocal microscopy was used to examine GFP and m-Cherry fluorescence (LSM 880; Zeiss, China).

### Yeast strains and vector manipulation

The yeast strains utilized in this study were (1) Zn^2+^ uptake deficient mutant zrt1/zrt2 (ZHY3; MAT; ade6; can1; his3; leu2; trp1; ura3; zrt1::LEU2; zrt2::HIS3) and (2) Zn^2+^ accumulation defective and high [Zn^2+^] sensitive mutant zrc1/cot1 (BY4741) (Lin et al., 2008).

The yeast expression vector, pFL61 (Minet et al., 1992), was used to transform the zrt1/zrt2Δyeast mutants, with *AtZIP7* serving as a positive control (Milner et al., 2013). *CaMTP* from pUC57 was digested using NotI. The digested product was subsequently ligated into the NotI site of pFL61, downstream of the phosphoglycerate kinase (PGK) promoter. The pFL61 vector harboring the insert in the correct orientation was identified *via* dual digestion of the plasmid with NotI and EcoRI.

The yeast expression vector pYUL2 was used to transform Δzrc1/cot1, in which *Medicago truncatula* MTP3 (*MtMTP3*) served as a positive control. The *CaMTP* coding sequence was ligated between the BamHI and HindIII sites downstream of the alcohol dehydrogenase1 (ADH1) promoter in pYUL2 following amplification with the primers “*CaMTP*-pYUL2-F” and “*CaMTP*-pYUL2-R” (Table 1).

### Yeast transformation and phenotypic analysis

Following the Yeastmaker Yeast Transformation System’s instructions, competent yeast cells were prepared and transformed (Takara). Following the directions on the Yeast Plasmid Extraction kit, plasmids were extracted and purified from the yeast cells (Solarbio, China). By screening the minimal synthetically defined (SD) base medium with -Ura DO Supplement (SD/-Ura; Takara) and PCR-based verification of the predicted inserts, yeast cells carrying the appropriate plasmid were identified.

Similar-sized yeast colonies were selected from the SD/-Ura selective medium and grown in 5 ml 1× Yeast Extract Peptone Dextrose medium (YPD) for 12–14 h at 250 rpm at 30°C. YPD contains 1% yeast extract, 2% peptone, and 2% dextrose. The pellets were twice rinsed with sterile water after the cultures were centrifuged at 700 g for 5 min. An OD600 of 1 was achieved by dissolving the yeast pellets in sterilized water and adjusting the volume. To create solutions with an OD600 of 0.1, 0.01, 0.001, and 0.0001, the cultures were diluted. A volume of 10 μl of these solutions were spotted onto solid full-nutrient medium or medium used to test Zn^2+^ and Fe^2+^-dependent growth phenotypes.

The positive and negative controls employed for the Zn^2+^ uptake-defective yeast mutant zrt1zrt2 were pFL61-AtZIP7 and pFL61 empty vector, respectively. The YPD medium is referred to as a full-nutrient medium. The YPD medium was added with 50 M ZnCl2 and 1 mM EDTA to investigate the Zn^2+^-deficient growth phenotype zrt1zrt2 (pH 6.5). The positive and control samples were pYUL2-MtMTP3 and the empty vector pYUL2, respectively, for assessing the growth phenotype of the Zn^2+^-storage-defective and excess Zn^2+^-sensitive yeast mutant zrc1cot1. The Zn^2+^-excess growth media was the YPD medium supplemented with 7 mM ZnSO_4_.

### Statistical analysis

One-way ANOVA and Tukey’s HSD test was used to examine the effects of the Zn treatments on plant dry weight, metal ion concentration, photosynthetic pigment levels, photosynthesis rate, and activity of antioxidant enzymes. Treatment effects were considered to be statistically significant at *p* < 0.05. Statistical analyses were performed in R version 3.6.1 (R Core Team, 2018).

## Results

### Effects of Zn treatments on plant growth

**Figure 1:**
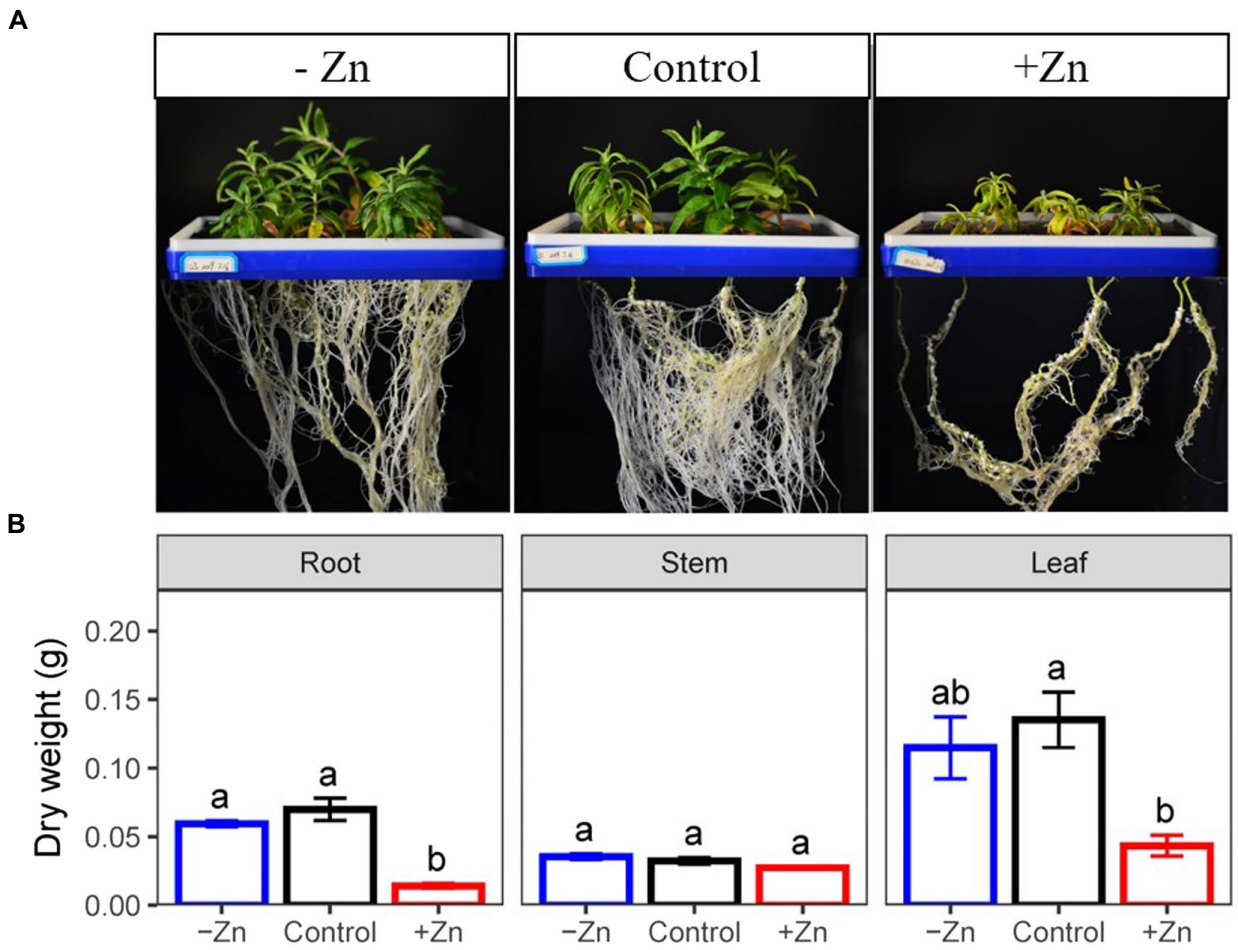
**(A)** Root, stem, and **(B)** leaf dry weight of *Ceratoides arborescens* subjected to Zn-deficient (0 mM L^−1^), control (0.015 mM L^−1^), and Zn-excess (0.15 mM L^−1^) conditions. Different lowercase letters indicate significant differences among treatments (*p* < 0.05).

At the end of the experiment, we found that Zn-excess inhibited the growth of *C. arborescens*, displaying retarded lateral root growth and leaf chlorosis (Figure 1A). Excessive Zn significantly decreased the root and leaf dry weight by 79.8 and 67.9% compared to the control, respectively (Figure 1B). However, Zn-deficient treatment did not affect the organic dry weight of *C. arborescens* (Figure 1B).

### Effects of Zn treatments on metal ion concentration

Zn-excess treatment significantly decreased Ca and Fe levels but increased Zn levels in the roots and shoots (Table 2). Compared to the control, the concentration of Zn increased by 4.6 times for roots, 15 times for stems, and 18 times for leaves under Zn-excess. Meanwhile, the concentration of Fe in the roots, stems, and leaves decreased by 74.5, 26.6, and 62.5%, respectively, and the concentration of Ca in the roots, stems, and leaves decreased by 39.6, 25.1, and 41.2%, respectively, under Zn-excess conditions. However, Zn-deficient treatment does not affect the concentration of Zn in plants. The concentration of Fe in stems increased by 1.14 times, while the concentration of Ca in leaves decreased by 41.4%.

**Table 2:** Zn, Fe, and Ca concentration in the root, stem, and leaf of *Ceratoides arborescens* subjected to Zn-deficient (0 mM L^−1^), control (0.015 mM L^−1^), and Zn-excess (0.15 mM L^−1^) treatments.

Treatment		−Zn	Control	+Zn
Zn concentration (μg mg^−1^)	Root	0.276 ± 0.038^b^	0.406 ± 0.118^b^	2.272 ± 0.074^a^
	Stem	0.092 ± 0.011^b^	0.075 ± 0.007^b^	1.227 ± 0.002^a^
	Leaf	0.083 ± 0.004^b^	0.066 ± 0.010^b^	1.259 ± 0.10^a^
Fe concentration (μg mg^−1^)	Root	5.084 ± 0.387^a^	4.543 ± 0.607^a^	1.158 ± 0.267^b^
	Stem	0.105 ± 0.017^a^	0.049 ± 0.002^b^	0.036 ± 0.007^b^
	Leaf	0.065 ± 0.004^a^	0.064 ± 0.001^a^	0.024 ± 0.001^b^
Ca concentration (μg mg^−1^)	Root	6.104 ± 0.237^a^	6.796 ± 0.470^a^	4.104 ± 0.102^b^
	Stem	11.062 ± 0.660^ab^	11.888 ± 0.614^a^	8.903 ± 0.451^b^
	Leaf	9.477 ± 1.232^b^	16.176 ± 0.152^a^	9.508 ± 0.705^b^

Different lowercase letters indicate significant differences among treatments (*p* < 0.05).

### Effects of Zn treatments on chlorophyll contents and photosynthesis activity parameters

**Figure 2:**
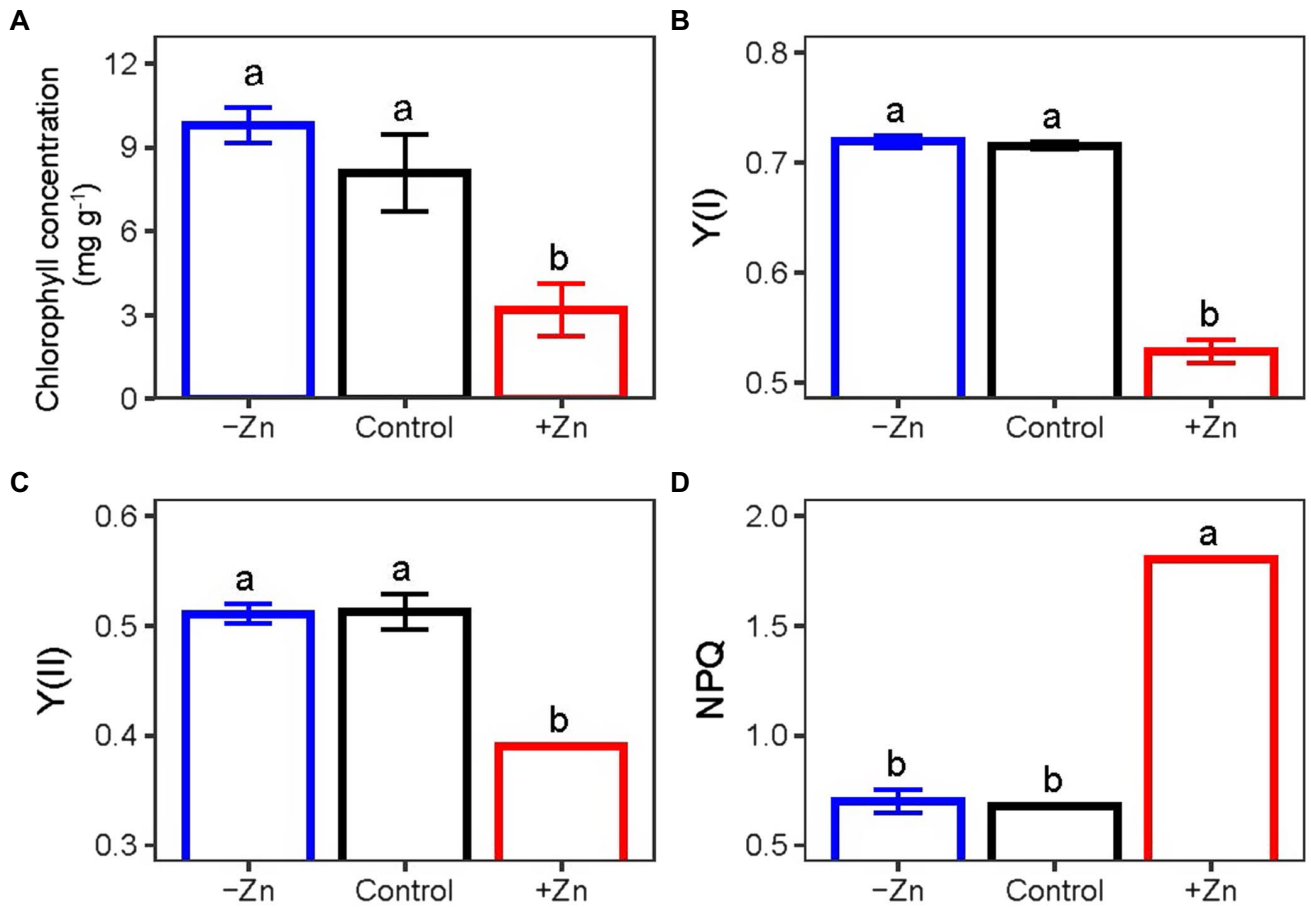
**(A)** The chlorophyll, **(B)** Y(I), **(C)** Y(II), and (**D**) NPQ levels of *C. Arborescens* under Zn-deficient (0 mM L^−1^), control (0.015 mM L^−1^), and Zn-excess (0.15 mM L^−1^) conditions. Different lowercase letters indicate significant differences among treatments (*p* < 0.05).

Treatment with high levels of Zn significantly influenced the chlorophyll contents and photosynthesis activity parameters. On average, Zn-excess treatment decreased the levels of chlorophyll (Figure 2A), Y(I) (Figure 2B), and Y(II) (Figure 2C) of *C. arborescens* by 30.6, 26.2, and 23.8%, respectively. Zn-excess treatment increased the NPQ level by 1.6 times (Figure 2D). However, Zn-deficient treatment did not affect the levels of chlorophyll, Y(I), Y(II), or NPQ of *C. arborescens* (Figure 2).

### Assessment of lipid peroxidation and the activity of antioxidant enzymes

**Figure 3:**
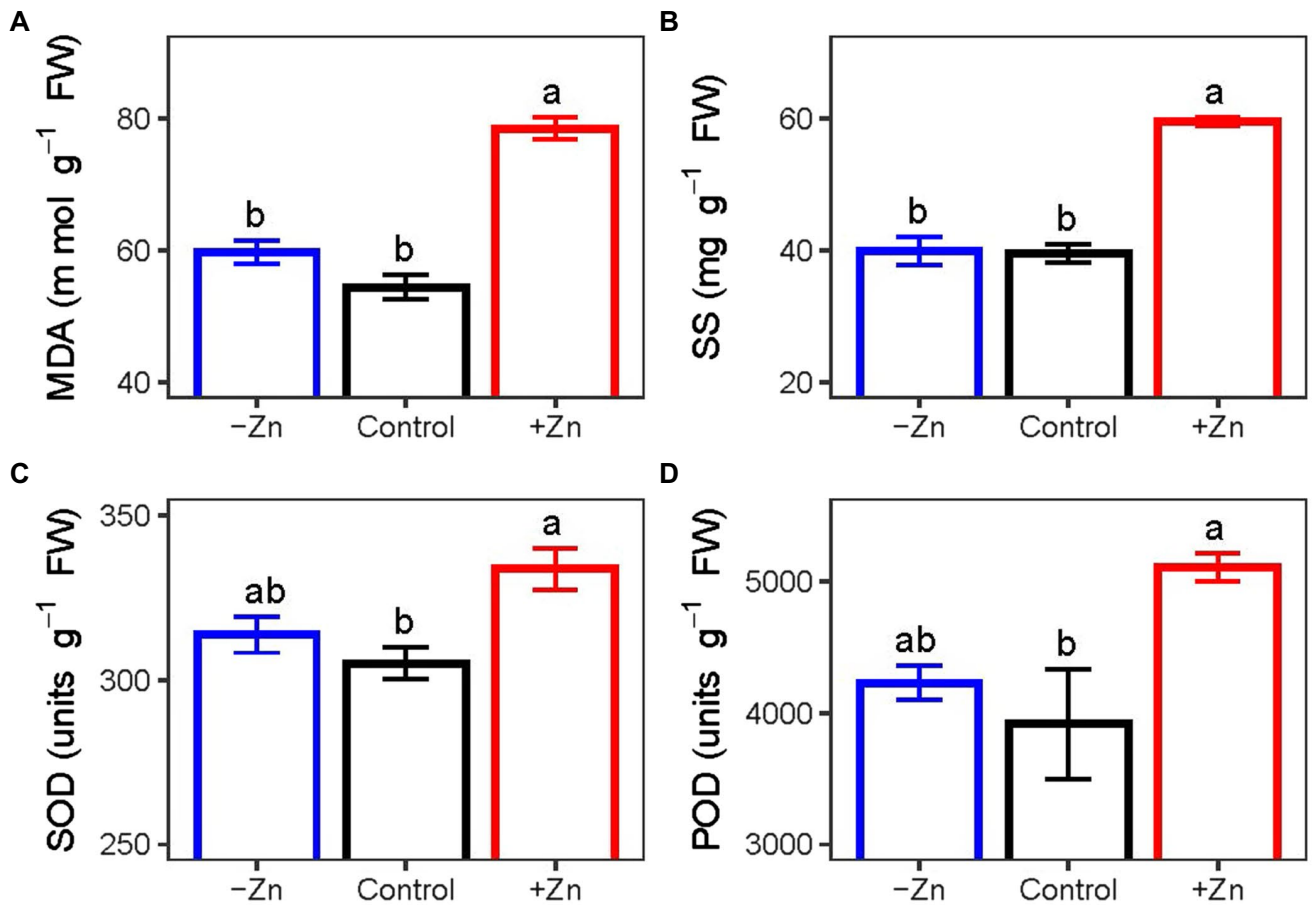
The concentrations of (**A**) MDA and (**B**) SS and activities of (**C**) SOD and (**D**) POD of *C. arborescens* subjected to Zn-deficient (0 mM L^−1^), control (0.015 mM L^−1^), and Zn-excess (0.15 mM L^−1^) conditions. Different lowercase letters indicate significant differences among treatments (*p* < 0.05).

Under excess levels of Zn, the concentrations of (A) MDA and (B) SS and the activities of (C) SOD and (D) POD in the roots of *C. arborescens* increased significantly. Compared to control, MDA concentration, SS concentration, SOD activity, and POD activity in the roots subjected to Zn-excess conditions were increased by 44.3, 50.7, 9.4, and 27.1%, respectively. However, Zn deficiency did not affect the activities of POD and SOD or the concentrations of MDA and SS (Figure 3).

### Identification of a *Ceratoides arborescens*-specific Zn transport protein-encoding transcript

**Figure 4:**
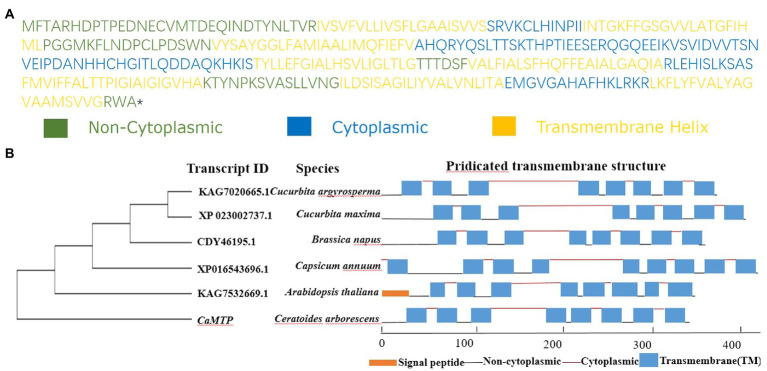
(**A**) Protein sequence of *CaMTP*. (**B**) Phylogenetic and structure comparison of *CaMTP* with other similar plant protein sequences. Different protein structure components are illustrated with different colors as indicated.

We analyzed the root transcriptome of *C. arborescens*, and discovered 39 distinct start codon-containing transcripts that may encode Zn-regulated transport proteins (Supplementary Table S1). We selected the full-length transcript DN154722 by assessing its sequence integrity and encoding protein structure prediction. We referred to it as *CaMTP*, while in Gene Ontology, it is referred to as “zinc II ion transport” (Figure 4A). With 80% residue identity and coverage, proteins from various species are regarded as homologs (Yu et al., 2004). Considering this, we could not identify any homolog of *CaMTP* in the NCBI protein database, where “Organism Optional” was *Dicotyledoneae*; instead, five proteins from five different plant species were identified with approximately 86% query coverage and 37% identity with that of *CaMTP*. Three of the five proteins had similar structures to that of *CaMTP* (Figure 4B).

### *CaMTP* localizes in the ER

**Figure 5:**
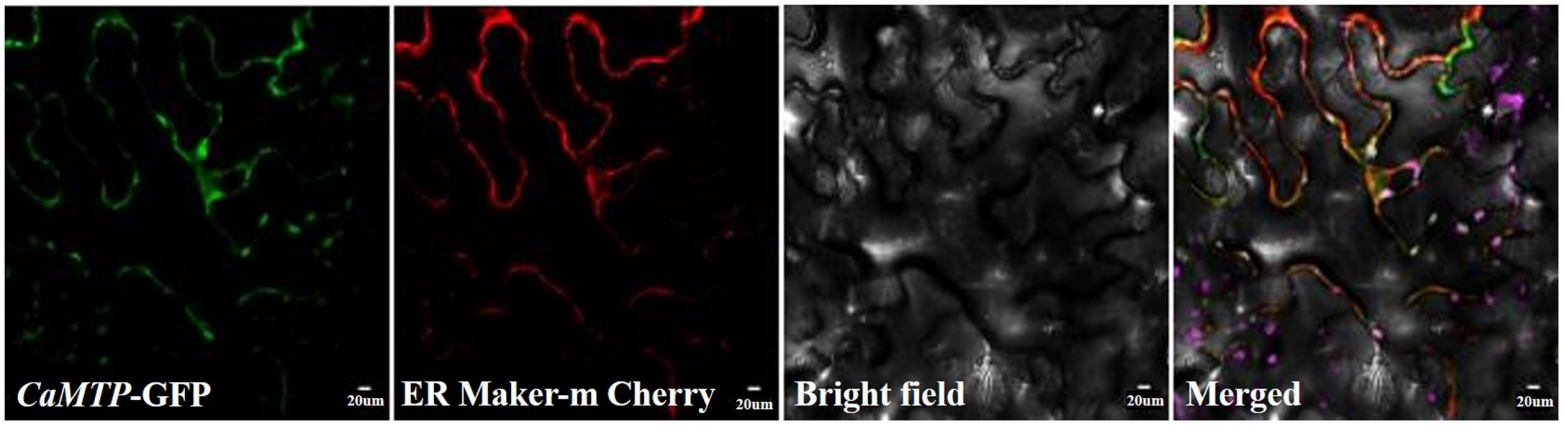
*CaMTP*-GFP and the ER marker ER-mCherry were transiently expressed in tobacco epidermal tissue and observed using conventional confocal microscopy.

To determine the subcellular localization of *C. arborescens* proteins in tobacco plant cells, we used the constructs expressing *CaMTP*-GFP fusion proteins. As shown in Figure 5, the *CaMTP*-GFP fusion protein showed strong co-localization with the ER marker ER-mCherry under a confocal laser scanning microscope, indicating that *CaMTP* is located in the ER.

### *CaMTP* complements a Zn^2+^ sequestration-deficient yeast mutant

**Figure 6:**
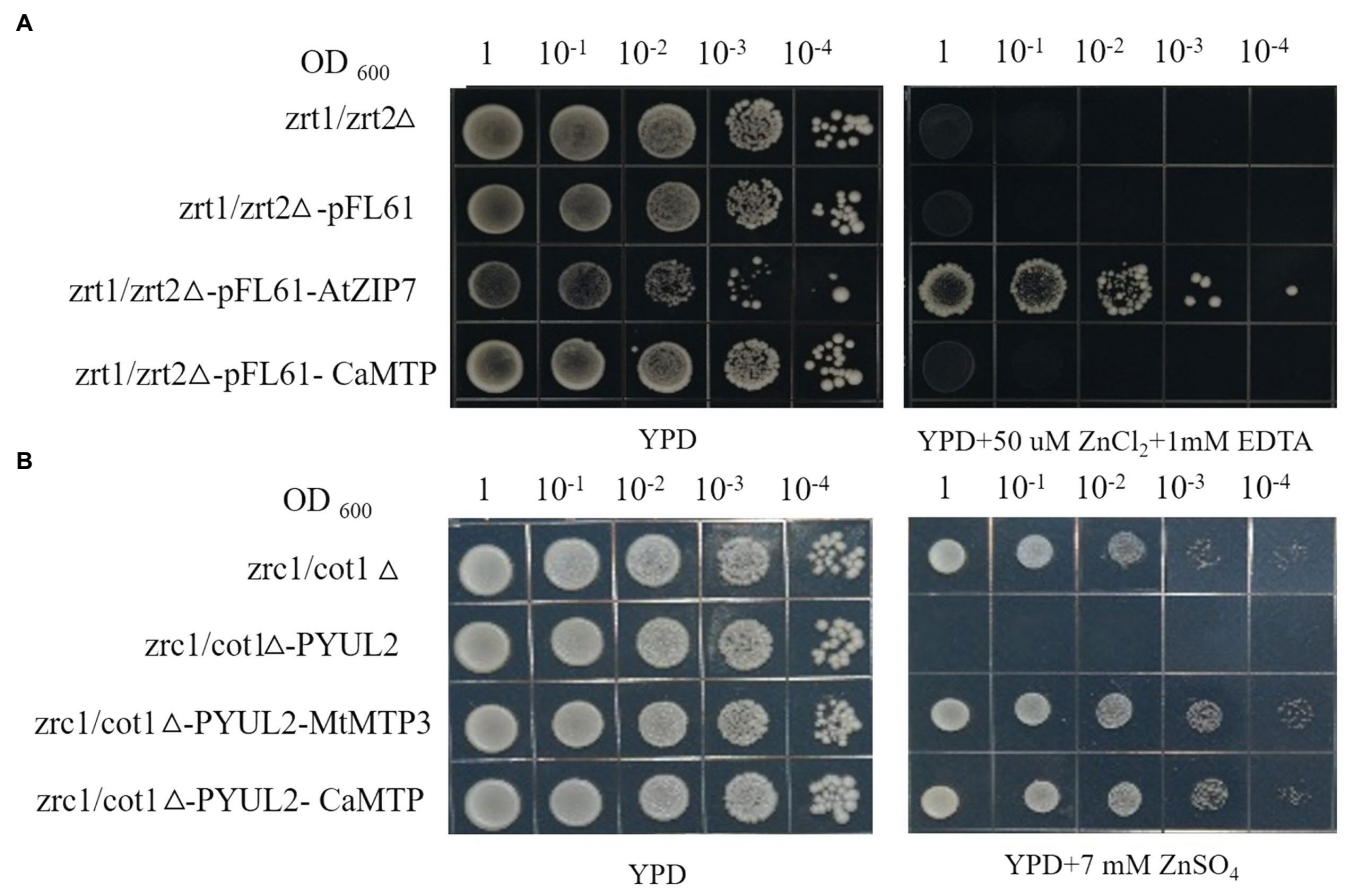
Complementation of yeast Zn^2+^ transport mutants with *CaMTP*. (**A**) The yeast cultures were spotted in full-nutrient YPD medium or Zn^2+^-deficient medium with EDTA. (**B**) The yeast cultures were spotted in full-nutrient YPD medium or Zn^2+^ excess medium with 7 mM ZnSO_4_.

Although AtZIP7 was utilized as a positive control, it did compliment the Zn^2+^-dependent growth of the yeast mutant zrt1zrt2, and neither did *CaMTP* (Figure 6A). In the yeast mutant zrc1cot1, which is susceptible to high Zn^2+^ levels, we transiently expressed *CaMTP*. Similar to the positive control, MtMTP3, *CaMTP* expression halted the mutant’s development when Zn^2+^ levels were excessive (Figure 6B).

## Discussion

Zn is a mineral involved in several physiological and biochemical processes, and its deficiency or excess interferes with the optimal growth of plants (Tiong et al., 2015; Bhantana et al., 2021; Shinozaki and Yoshimoto, 2021; Al Jabri et al., 2022). In our study, high Zn supply has negative effects on the plant, as demonstrated by the decline in the dry weight of leaves and roots as well as leaf chlorosis and abnormally brown roots. These results are consistent with previous findings. For example, growth was restricted when *Moso bamboo* was treated with elevated levels of Zn due to the changes in the root structure (Liu et al., 2014). Growth inhibition and chlorosis in tomato leaves were observed when exposed to excess Zn (Cherif et al., 2010). Compared with these results, *Juncus acutus* could survive and displayed no toxicity symptoms when subjected to Zn at concentrations as high as 100 mM L^−1^, a concentration considerably greater than those applied in our experiments (Mateos-Naranjo et al., 2014). This discrepancy in Zn tolerance was associated with the capacity to accumulate Zn in roots (at concentrations up to 2,500 mg kg^−1^) and largely avoiding its transport to tillers. Although the Zn concentration of *C. arborescens* increased 18 times in the leaves and 15 times in the stems, while only 4.6 times in the roots, but the concentration of zinc in roots is nearly twice that of aboveground parts. Furthermore, the root was the most responsive to Zn in terms of dry weight reduction (79.8%), when compared to that of the leaf (67.9%) and stem (15.6%). Roots are seriously affected by heavy metals may be due to that Zn exposure could induce significant modifications in root architecture either by decreasing root-specific superficial area (Balafrej et al., 2020) or lignification of plant root tips (Glińska et al., 2016), thereby lowering the capacity of water and nutrient absorption, impacting plant growth and thus the plant dry weight negatively (Scheid et al., 2017).

We also found that excessive Zn inhibited photosynthesis by reducing Y(I) (Photosystem I; PSI) and Y(II) (Photosystem II; PSII) since Zn toxicity negatively impacts plant photosynthesis and thus decreases plant biomass (Sagardoy et al., 2010; Kaur and Garg, 2021). Similar results were reported in the *Datura* species grown at higher Zn concentrations (Vaillant et al., 2005). PSII efficiency declined with the increase in Zn concentration (Dhir et al., 2011). Under metal stress, an imbalance between light absorption and light energy usage results in a surplus of light energy, causing an increase in NPQ and a decrease in the PSII efficiency (Sárvári, 2005). The negative impact of high Zn concentration on the integrity of the plasma membrane and photosynthetic electron transport impeded photosynthesis and reduced membrane permeability (Vijayarengan and Mahalakshmi, 2013). Furthermore, chlorophyll is important for plant photosynthesis (Croft et al., 2017). The reduction of chlorophyll content possibly declined the number of PSI units along with activity (Kudoh and Sonoike, 2002). Zn interferes with chlorophyll biosynthesis either by substituting for the Fe/Mg ions and inhibiting key biosynthetic enzymes, mainly δ-aminolevulinic acid dehydratase and protochlorophyllide reductase, or through the oxidative damage of chloroplasts (Marichali et al., 2016). Excess Zn reduced chlorophyll concentration and caused leaf chlorosis in our study, which was in line with the findings based on the mustard plant (Cherif et al., 2010; Khan and Khan, 2014). Thus, the reduction in chlorophyll concentration in leaves could be linked to a decrease in Fe content (Hu et al., 2017). However, the chlorophyll synthesis in tobacco leaves was not significantly inhibited under Zn stress, as the expression of other important proteins did not significantly change (Zhang et al., 2020).

Moreover, one of the consequences of heavy metals exposure in plants is lower absorption of other mineral elements(Kabata-Pendias, 2000; Kaur and Garg, 2021; Zaheer et al., 2022). In this respect, our assessment of mineral nutrients showed that high Zn caused a nutritional imbalance in *C. arborescens* plants, and Ca and Fe concentrations in roots and leaves were lower than those in the control plants. This fact indicates that high Zn supply impacts the transport of other elements and the uptake of *C. arborescens*, because the transporter of divalent metal cations, such as Zn Fe Mn, generally exhibits broad substrate specificity (Vigani et al., 2018; Kabir et al., 2021). Previous studies have also suggested that Zn transport proteins can transport Fe or Cu ions (Sinclair and Krämer, 2012; Sinclair et al., 2018). The interactions between Zn-Ca and Zn-Fe have also been previously described (Kabata-Pendias, 2000). The reduction of Ca concentration might be involved in the regulation of carbohydrate metabolism under Zn stress (Zhang et al., 2017). The reduction of Fe concentration might be attributed to competing sites being the direct cause of enthalpy among metal ions (Li et al., 2020).

Metal toxicity exacerbates ROS production, which damages lipids, proteins, and nucleic acids and disrupts metabolism (Miller et al., 2008; Zaheer et al., 2020b). However, plants evolved sophisticated antioxidant defense mechanisms to counteract and undo the harm caused by ROS, including activities of various antioxidant enzymes such as SOD and POD (Rout and Das, 2003; Kumar Tewari et al., 2008; Javed et al., 2020). In this study, higher SOD and POD activities were observed in the seedlings of *C. arborescens* under excess Zn. Our findings are concordant with the results of *Spinacia oleracea, Handroanthus impetiginosus* and *Tabebuia roseoalba* (Gai et al., 2017; Zaheer et al., 2020a). Excessive Zn levels can promote MDA production in plants based on enhanced lipid peroxidation *via* excessive generation of free radicals (Cherif et al., 2010). Our results revealed that MDA concentrations in shoots under Zn-excess conditions were 44.3% higher compared to the control, a distinction from the results of the mulberry plant under Zn-deficient conditions where the increase observed in Zn-excess plants was not significant (Kumar Tewari et al., 2008). Additionally, to regulate their response to heavy metal stress, plants could increase the synthesis and storage of osmolytes like soluble proteins, soluble carbohydrates, and free proline (Sharma and Dietz, 2006). Herein, we discovered that roots’ SS levels were significantly elevated in response to Zn stress, suggesting a defense mechanism against Zn toxicity. SS functions as signal molecules in sugar sensing and signaling systems in addition to their regular osmoprotective roles of stabilizing cell membranes and preserving turgor (Wu et al., 2011).

Zn deficiency causes leaf bronzing, growth stunting, delayed maturity, and plant yield loss (Broadley et al., 2007). Conversely, Zn deficiency did not affect the growth of *C. arborescens*. These results were corroborated by the plant biomass amount and the unchanged concentration of chlorophyll under Zn deficiency. In *Zea mays* L. cv. PAC (Zn tolerant line), Zn deficiency did not significantly affect shoot height or weight, indicating that PAC can resist Zn deficiency, which was corroborated by the reduction of Zn concentration in both root and shoot in DAC (Zn sensitive line), but the concentration in PAC was maintained under Zn deficiency (Khatun et al., 2018). The decrease in root and shoot Zn concentration in *Ratan* (Kabir et al., 2021) suggests that Zn homeostasis cannot be maintained under Zn deficiency by these genotypes. In this study, we also found that the Zn concentration in both shoots and roots did not change. In contrast, Zn deficiency decreased the contents of Ca in leaves. A previous study also indicated that autophagy resupplies can alleviate the Zn deficiency state and promote optimal growth when environmental Zn is unavailable (Shinozaki and Yoshimoto, 2021). Furthermore, SOD and POD are involved in plant stress tolerance (Kumar Tewari et al., 2008). Herein, we found that the activities of POD and SOD did not change under Zn deficiency, suggesting that *C. arborescens* was not stressed under Zn deficiency. In contrast, increased activities of SOD and POD have been reported in response to Zn deficiency in tomatoes (Kabir et al., 2021), while decreased SOD activities were recorded in *Coffea arabica* (dos Santos et al., 2019). This also reflects that different species respond differently to Zn deficiency. We reasoned that the high Zn content in the seeds (Supplementary Table S2) could compensate for seedling growth in *C. arborescens*; hence it grows well in Zn deficiency.

Identifying novel proteins to increase crop stress tolerance in wild plants, particularly those acclimated to severe settings, is promising (Yang et al., 2018). The rapid advancement of high-throughput sequencing technologies (Bräutigam and Gowik, 2010) and the identification of yeast mutants (Zhao and Eide, 1996; Lin et al., 2008) have tremendously aided in the discovery of new genes and proteins. Our study reports the discovery of a novel protein in *C. arborescens*, which showed strong tolerance against Zn stresses. Transcript profiling and comparison of deduced protein sequences identified a transmembrane transcript, *CaMTP*, whose significant homolog was not found in other plants. Contrary to the findings for all previously described members of the ZIP family, *CaMTP* has no passive transport activity for Zn^2+^ (Milner et al., 2013). We transiently expressed *CaMTP* in yeast mutant zrc1cot1Δ, which is sensitive to excess Zn^2+^ (Lin et al., 2008). The expression of *CaMTP* rescued the growth of the mutant under high Zn^2+^ conditions, just like the positive control, *MtMTP3* (Zhao et al., 2018). Members of this protein family typically transport metal out of the cytosol (Krämer et al., 2007; Zhao et al., 2018). We observed that *CaMTP* is located in the ER, similar to MTP2, which may mediate the uptake of cytoplasmic Zn^2+^ into ER (Sinclair et al., 2018). The Arabidopsis CDF family proteins Metal Tolerance Protein 12 (*AtMTP12*) and 5 (AtMTP5) are co-located on the Golgi membrane, and interact with each other (Fujiwara et al., 2015). The cucumber protein *CsMTP5* is also located on the Golgi apparatus (Migocka et al., 2018). This fact indicates that *CaMTP* has characteristics of the CDF family Zn transporter, while the transport function of other metals is not clear.

In conclusion, our study examined the tolerance of Zn and identified a specific Zn transport protein-encoding transcript in *C. arborescens*. The results showed that excess Zn drastically decreased the biomass and induced chlorosis and growth defects in plants. Excess Zn increased the concentration of Zn but decreased that of Fe and Ca in the roots and shoots, along with the content of *C. arborescens*, thereby decreasing photosynthesis activity. The increased MDA and SS levels and POD and SOD activities may be part of a mechanism to resist high-level Zn stress. However, the shortage of Zn supply did not affect the growth of *C. arborescens*, suggesting that this forage could be established in the Zn-deficient area. Further, we identified a novel endoplasmic-localized protein named *CaMTP*, which complemented the increased sensitivity of a yeast mutant to excessive Zn. The Zn-deficiency tolerance mechanisms in plants are not fully known, to further understand Zn homeostasis in plants, the mechanisms related to Zn deficiency in *C. arborescens* should be investigated. Moreover, to further research *CaMTP,* the genetic transformation system of *C. arborescens* should be built.

## Data availability statement

The data presented in the study are deposited in the NCBI GenBank (https://www.ncbi.nlm.nih.gov/). The associated BioProject, Bio-Sample and SRA numbers were PRJNA870161, SAMN30353563- SAMN30353571, and SRR21095981-SRR21095989 respectively.

## Author contributions

FM designed the study. XL conducted the experiments, analyzed the data, and wrote the manuscript. LZ guided the experiment. HR and XW revised the manuscript. All authors contributed to the article and approved the submitted version.

## Funding

This research was supported by international cooperation between China and the European Union Center (2017YFE0111000).

## Acknowledgments

We would like to thank Zhi Qi for providing the experimental site and equipment, Wanjie Chen for statistical advice, and Editage (www.editage.com) for English language editing.

## Conflict of interest

LZ is employed by M-Grass Ecology and Environment (Group) Co., Ltd.

The remaining authors declare that the research was conducted in the absence of any commercial or financial relationships that could be construed as a potential conflict of interest.

## Publisher’s note

All claims expressed in this article are solely those of the authors and do not necessarily represent those of their affiliated organizations, or those of the publisher, the editors and the reviewers. Any product that may be evaluated in this article, or claim that may be made by its manufacturer, is not guaranteed or endorsed by the publisher.

## Supplementary material

The Supplementary material for this article can be found online at: https://www.frontiersin.org/articles/10.3389/fpls.2022.976311/full#supplementary-material

## Footnotes

^1^https://blast.ncbi.nlm.nih.gov/Blast.cgi (Accessed July 28, 2022).

^2^http://www.ebi.ac.uk/interpro/ (Accessed July 28, 2022).
