# Supplementary material for: Zinc toxicity response in Ceratoides arborescens and identification of CaMTP, a novel zinc transporter
Source: Front Plant Sci. 2022 Sep 6;13:976311. doi: 10.3389/fpls.2022.976311 (PMC9505901; doi:10.3389/fpls.2022.976311)
Supplement: Supplementary file 1 [file Data_Sheet_1.docx]

Supplementary Material

# Supplementary Tables

# Table S1: Thirty-nine transcripts putatively encoding zinc/ion-regulated transporter proteins

| Transcript ID | Annotation | Transcript ID | Annotation |
| --- | --- | --- | --- |
| DN161460 | iron ion transmembrane transporter activity | DN156316 | Zinc transporter ZIP9 |
| DN169412 | ferrous iron transmembrane transporter activity | DN160458 | zinc ion transmembrane transporter activity |
| DN170650 | iron ion transport | DN161883 | Zinc transporter ZIP9 |
| DN172052 | iron ion transport | DN163936 | Zinc transporter ZIP8 |
| DN173301 | iron ion transmembrane transporter activity | DN168558 | zinc transporter 3-like |
| DN174140 | iron ion transmembrane transporter activity | DN169219 | zinc II ion transmembrane transport |
| DN176093 | iron ion transmembrane transport | DN170095 | Zinc transporter 1 |
| DN178699 | zinc II ion transport；iron ion transport | DN170165 | Zinc transporter 8 |
| DN180533 | iron ion transport | DN170205 | zinc II ion transmembrane transport |
| DN183905 | ferric iron transport | DN170252 | zinc ion transmembrane transporter activity |
| DN162867 | intracellular sequestering of iron ion | DN172107 | Zinc transporter 10 |
| DN154722 | Fe(2+) transport protein 3/zinc II ion transport | DN172419 | Zinc transporter 9 |
| DN173090 | iron ion transmembrane transporter activity | DN172922 | zinc ion transmembrane transporter activity |
| DN164529 | ZIP family transporter | DN175877 | zinc ion transmembrane transporter activity |
| DN177758 | bZIP transcription factor 60 | DN176213 | zinc II ion transmembrane transport |
| DN177894 | zinc II ion transmembrane transport | DN180230 | zinc ion transmembrane transporter activity |
| DN177935 | zinc II ion transmembrane transport | DN180800 | zinc II ion transmembrane transport |
| DN178722 | zinc II ion transmembrane transport | DN181866 | zinc ion transmembrane transporter activity |
| DN180011 | zinc II ion transmembrane transport | DN182700 | zinc ion transmembrane transporter activity |
| DN184150 | zinc ion transmembrane transporter activity |  |  |

**Table S2**: Zn concentration in the seeds and bracts of *C. arborescens.* Different lowercase letters indicate significant differences among treatments (*P* < 0.05).

| **Organic** | **Zn concentration (µg mg ^-1^)** | **groups** |
| --- | --- | --- |
| seeds | 0.0491±0.0004 | a |
| bracts | 0.0157±0.0010 | b |
